# An argument against the focus on Community Resilience in Public Health

**DOI:** 10.1186/1471-2458-14-62

**Published:** 2014-01-21

**Authors:** Peter Allmark, Sadiq Bhanbhro, Tom Chrisp

**Affiliations:** 1 Centre for Health and Social Care Research, Sheffield Hallam University, 32 Collegiate Crescent, Sheffield S10 2BP, England; 2 Arc Research and Consultancy Ltd, 15 Winsford Road, Sheffield S6 1HT, England

**Keywords:** Public health, Community, Resilience, Social determinants, Social class, Community networks, Poverty, Community integration

## Abstract

**Background:**

It has been suggested that Public Health professionals focus on community resilience in tackling chronic problems, such as poverty and deprivation; is this approach useful?

**Discussion:**

Resilience is always i) of something ii) to something iii) to an endpoint, as in i) a rubber ball, ii) to a blunt force, iii) to its original shape. “Community resilience” might be: of a neighbourhood, to a flu pandemic, with the endpoint, to return to normality. In these two examples, the endpoint is as-you-were. This is unsuitable for some examples of resilience. A child that is resilient to an abusive upbringing has an endpoint of living a happy life despite that upbringing: this is an as-you-should-be endpoint. Similarly, a chronically deprived community cannot have the endpoint of returning to chronic deprivation: so what is its endpoint? Roughly, it is an as-you-should-be endpoint: to provide an environment for inhabitants to live well. Thus resilient communities will be those that do this in the face of challenges. How can they be identified?

One method uses statistical outliers, neighbourhoods that do better than would be expected on a range of outcomes given a range of stressors. This method tells us *that* a neighbourhood is resilient but not *why* it is. In response, a number of researchers have attributed characteristics to resilient communities; however, these generally fail to distinguish characteristics of a good community from those of a resilient one. Making this distinction is difficult and we have not seen it successfully done; more importantly, it is arguably unnecessary.

There already exist approaches in Public Health to assessing and developing communities faced with chronic problems, typically tied to notions such as Social Capital. Community resilience to chronic problems, if it makes sense at all, is likely to be a property that emerges from the various assets in a community such as human capital, built capital and natural capital.

**Summary:**

Public Health professionals working with deprived neighbourhoods would be better to focus on what neighbourhoods have or could develop as social capital for living well, rather than on the vague and tangential notion of community resilience.

## Background

The idea that community resilience can contribute to Public Health has developed first, in relation to disaster preparedness [1] and, more recently, in relation to longer term health threats, such as poverty and unemployment [2-4]. This paper’s focus is on this second use; it queries the underlying idea that the health of communities could be maintained or improved by drawing upon and fostering community resilience. Our discussion is based in a critical review of the literature that focused on the term ‘resilience’ and cognates restricted to its application to the term ‘community’ and cognates. As such, we did not seek literature focused on other uses, such as individual psychological resilience except insofar as it was relevant to our focus. The details of the search are available from the first author of this paper (PA).

One attraction of the resilience approach is that it is asset rather than deficit-based. Kretzmunn and McKnight [5] suggest two paths to community development, the traditional needs-driven approach and an alternative capacity-focused or asset-based one. The former focuses on community needs, problems and deficiencies. By contrast, asset-based approaches focus on what is present in the community, the capacities of its residents and workers, and the associational and institutional base of the area. As well as being more positive than the deficit-based glass-half-empty approach, the asset based approach might be cheaper and more successful as it draws upon and develops positive factors already present or potential in the community.

Resilience is also a concept that fits with the political agenda of the current (2013) UK Government to pull back the State and foster independence. One criticism of having a large Welfare State is that it encourages dependence in those who receive it and discourages community virtues of charity and altruism: the criticism has been made in relation to countries noted for large Welfare States, such as Sweden [6] as well as to the UK [7,8] and to the United States [9], which has far smaller welfare provision from the State. The present UK Government would prefer community welfare to be looked after by the community itself; this is sometimes described as the big-society agenda [10].

For the notion of community resilience to be useful to Public Health practitioners, they must know how it is to be identified, assessed and fostered. Such practitioners are the intended audience of this paper; hence the discussion is structured round those three themes. It begins with the meaning of the terms, resilience and community.

## Discussion

### Definition

#### a) Definition of Resilience

The term ‘resilience’ is of 17th century origin and was originally used in physics to denote the ability of an object to absorb and then release energy when deformed elastically [11]. Thus a rubber ball is resilient; subject to a blunt force it will deform and then rebound to its original shape; a crystal ball will shatter in the same circumstances and is thus not resilient. The resilience of a cannon ball is less obvious; however, although it takes a great force to deform it elastically, once this has happened, it does not release the energy and resume its original shape. As such, a cannon ball is not particularly resilient, although it is strong or deformation resistant.

‘Resilience’ seems to have begun use as a metaphor in the 19th century. The Oxford English Dictionary (OED) notes three now obscure metaphorical uses before setting out a fourth that is still used and is the one of interest in this paper [11]:

“The quality or fact of being able to recover quickly or easily from, or resist being affected by, a misfortune, shock, illness, etc.; robustness, adaptability.”

The OED gives four examples of its use ranging from 1857 to 2002; of these, three refer to individuals and one, from 1857, refers to a collective, the Scottish people. This metaphorical use was extended in the 1970s into the arena of ecology [12,13]. An early example is the resilience of the seas to oil spills. In various areas of engineering the term has been used to describe products, production systems and computer networks. A computer system with reliable back-up memory is resilient. In the social sciences the term has been used in economics to describe supply chains and organisations [14], and in psychology, to describe the capacity to resist factors conducive to mental illness; for example, trauma is associated with mental illness but most who suffer trauma come through without such illness [12,15-24]. Finally, as we have seen, the term has also been applied to communities to mean, roughly, the capacity of a community to rebound from events conducive to community dysfunction or breakdown [4,16,24-37].

In all these uses, resilience is the internal quality i) of something ii) to return to a state (such as equilibrium) iii) in the face of external challenge or adversity. In other words, resilience is of something, to something, to some endpoint. Table 1 compares some of the uses.

**Table 1:** Different uses of the term resilience

**Of what?**	**To what?**	**To what endpoint?**
Rubber ball	Blunt force	Form previous to blunt force
Ecosystem	e.g. Oil spill	Previous biodiversity
Organisation	e.g. Supermarket supply-chain problem	Previous supply of goods to customers
Individual psychology	e.g. Mugging	Mentally healthy life
Communities	e.g. Earthquake	Previous state of lifestyle for community members

Note that the example stressors in the table are acute, short-term shocks to the system. As such, the resilience of the system (such as the ball or the individual person) is marked by its capacity to return to normal: the endpoint, in other words, is as-you-were.

At this point, however, a distinction needs to be made that is, to our knowledge, new to the literature. It is that the as-you-were endpoint is unsatisfactory in relation to chronic problems, extended over time, such as a child growing up in an abusive household [38,39], or a community facing long-term poverty and unemployment [29,30,40-44]. The desirable endpoint for a child in an abusive household relates to what she becomes, not her starting point; it is not as-you-were but rather as-you-should-be. Roughly, we hope she becomes an adult with reasonable mental health who is able to function in society. It was the recognition that most individuals from apparently deprived backgrounds fared reasonably well as adults that was the origin of the use of the term ‘resilience’ in relation to people and in the asset-based approach to psychology [45].

This as-you-should-be endpoint requires a value judgement; how the system should be. In the example of the child growing up in an abusive household this seems reasonably straightforward; what she should be (amongst other things) is mentally well. But we would be less willing to judge an individual as resilient if, against the odds, she becomes a wealthy and successful criminal, even though she might judge herself as such. We tend to use other terms to denote the ability of individuals or things to carry on when we’d rather they didn’t. The ability of an individual or community to carry on with high levels of unhealthy behaviour in the face of pressure from public health professionals might be called stubborn rather than resilient, as might the ability of an organisation such as the Mafia to resist the efforts of law enforcement. This is not to say that the term ‘resilience’ is never used pejoratively, just that it is usually not. Table 2 summarises the new terminology introduced thus far.

**Table 2:** Summary of new terminology

**Endpoint**	Something’s resilience is a function of its ability to reach an endpoint having been subject to a stressor (or distorting force) that tends to move it away from that endpoint.
**As-you-were endpoint**	The paradigm endpoint is of an as-you-were type; that is, something’s resilience is the extent to which it can revert to the state it was in before being subject to the stressor (as with a rubber ball subject to a blunt force).
**As-you-should-be endpoint**	However the term ‘resilience’ is sometimes applied in cases where an as-you-were endpoint would not apply. For example, a child growing up in an abusive family would be deemed resilient if he developed into a well-balanced adult, not if he remained an abused child. Here it seems better to talk of an as-you-should-be endpoint.
**Acute and Chronic stressors**	A stressor is acute if its impact is fairly brief: examples include a rubber ball thrown against a wall and a community subject to an earthquake. It is chronic if its impact is long term: examples include a rubber ball stored long term under pressure and a community subject to chronic poverty.

Note that the judgements above of as-you-should-be resilience are of individuals or organisations. In such cases resilience seems to relate to good functioning; a resilient organisation or individual is able to function well in the face of difficulty. The notion of organisations having a function is straightforward. There is also an extensive philosophical literature on the notion of good human functioning. This has origins in Classical Greece but is still a live topic; the idea is that someone functions well when they live well [46]. By contrast, the basis in relation to community resilience is not clear because a community has no obvious function against which we can decide whether it is as-it-should-be. How do we decide what a community should be?

#### b) Definition of communities

To answer this question we need to discuss the notion of community itself. A commonly-used categorisation is between communities of [47]:

• Location, such as a neighbourhood;

• Culture, such as an ethnic group;

• Purpose, such as a political association.

Some communities will be combinations of these. For example, a Native American rights group living in a reservation combines all three. Indeed, much of the literature relating to resilient communities comes from the USA; in one example, Hispanic populations are noted as resilient to poverty [48]. However, this coincidence of a community of culture, the Hispanics, with a community of location, the Hispanic quarter, applies more clearly in North American than in European cities. In multi-ethnic cities in the UK, such as London or Birmingham, it is rare to find mono-ethnic neighbourhoods. As such, UK neighbourhoods are likely to share fewer of the ties of culture and purpose seen in US neighbourhoods. Such mono-ethnic neighbourhoods as there are will tend to be of the majority White population, thus lacking any sense of shared difference with wider society and thus of homogeneity within. In such neighbourhoods, people may feel they have little in common with each other and no sense of belonging to the area: they might feel stronger ties to a community within or outside of the neighbourhood based, for example, on ethnicity. This raises a problem for practitioners: when policy makers speak of resilient communities they tend to mean deprived neighbourhoods; but these combine the difficulty of knowing what we mean by resilience (because it is hard to attribute to localities an as-you-should-be endpoint) with a lack of sense of community held by people in the neighbourhood. Can this difficulty be overcome?

In the table above we gave the example of an earthquake as a stressor to a neighbourhood broken down by the three-question framework (of what, to what, to what endpoint?). Let us now extend this to take in three different types of community and two different types of stressor. This is illustrated in Table 3; note that the last two boxes show again the problem of defining endpoints for a resilient community.

**Table 3:** Community stressors and endpoints

***Of what (type of community)***	***E.g. To what? Acute A) or Chronic C) Stressors***	***Type of end***	***Endpoint***
Purpose e.g. political association	A) Database loss	As-you-were^1^	(To campaign for) political goals
	C) Ageing membership, loss of leader, internal argument		
Culture e.g. religion, ethnicity	A) Violent assault on religious Centre	As-you-were	Maintenance of religion, language and so on
	C) Young people exposed to dominant antithetical culture		
Location e.g. neighbourhood	A) Earthquake	As-you-were	Previous state of lifestyle for community members
	C) Poverty, unemployment	?	?

^1^There is room to dispute whether the endpoint of a political association is as-you-were (the activity of promoting political ends) or as-you-should-be (the state of achieving those ends). We have taken it to be the former as the political association would lose its purpose and, presumably, dissolve if its ends were achieved.

How might the two incomplete boxes be filled? In broad terms it seems reasonable to say that almost all people seek to live well, to flourish, to be happy and so on. And although there are huge differences in people’s picture of what a good life consists in, there are likely to be some common elements, such as having reasonably good health. These common elements are at the heart of the Capability Approach [49].

The Capability Approach developed from work in the measurement of economic progress. In standard approaches an economy is said to be progressing if its Gross National Product (GNP) increases. There are numerous problems with this: for example, a rising GNP is compatible with rising inequality or with lack of opportunity for groups in society, such as women or minority ethnic groups [50-52]. In contrast, under the Capability Approach what matters in assessing an economy is people’s opportunity for functionings, that is, what they can be and do. The to-be functionings are states such as being well nourished, being in a warm house, and being literate. The to-do functionings are activities such as travelling, voting, having a family life, having a rewarding job; all things we might associate with living well [52]. The opportunities for functioning are an individual’s capability. They come in sets: for example, an individual with a low-paid job might be able to earn enough to feed her family but at the price of being unable to spend time with the family. Central to the Capability Approach is that if an individual does not have within her set of choices an option in which all important to-do and to-be functionings are available, then her wellbeing is compromised. Most of the to-do and to-be functionings are unavailable to an individual in complete isolation, a Robinson Crusoe character. Human beings are dependent and interdependent social animals who can flourish a) only in communities and b) only in communities that provide a minimum level of social goods or, in other words, an adequate Capability set.

This gives a plausible endpoint for neighbourhoods. It is of the as-you-should-be type and is: to contribute positively to the inhabitants’ wellbeing (or Capability set). A good neighbourhood is one which does; a resilient one has the additional quality of being able to do so in the face of potentially undermining stressors, such as poverty. Table 4 below shows this by completing the gaps left on Table 3.

**Table 4:** Neighbourhood chronic stressors and endpoint

***Of what (type of community)***	***E.g. To what? Acute A) or Chronic C) Stressors***	***Type of end***	***Endpoint***
Location e.g. neighbourhood	C) Poverty, unemployment	As-you-should-be	Provides good environment for human functioning.

### Identification

**Figure 1:**
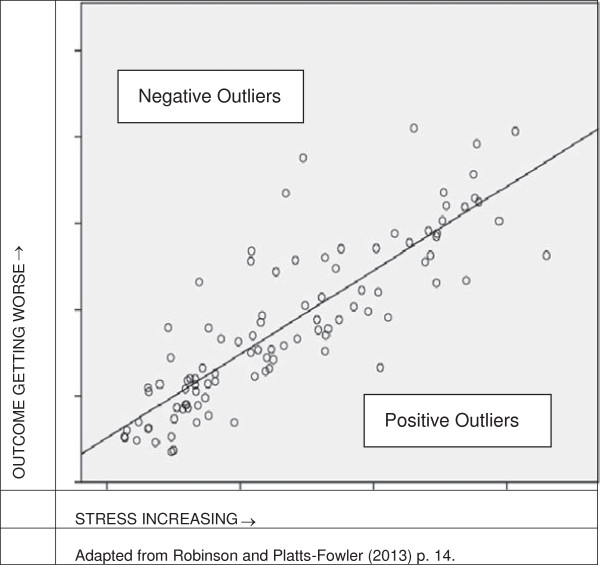
Identifying outlier neighbourhoods.

Let us turn now to the question of identifying communities’ contribution to wellbeing and their resilience. One such method involves identifying outliers, that is, looking for neighbourhoods that are doing better than would be expected in relation to certain outcomes given certain stressors. A simple model developed by Robinson and Platts-Fowler, delineates stressors on the basis of the readily available data on unemployment, income and deprivation; the model sets these against outcomes related to community safety and cohesion, health and wellbeing, and inclusion [53]. When this was done for the neighbourhoods of Sheffield, the following graph was obtained (Figure 1).

Each circle represents a neighbourhood. The neighbourhoods lying furthest out on the x-axis are the most deprived; those that lie beneath the line marking the average are doing relatively well whatever their level of deprivation. Hence we can say that those lying beneath the average line and furthest out on the x-axis are resilient. This method has a number of virtues: i) it is precise in defining the community, the stressors and the endpoints; ii) it uses easily available data and is thus transferable; and iii) its meaning is well presented graphically. It has at least two important limitations: first, the list of endpoints is constrained and from a Capability viewpoint is inadequate to show that a neighbourhood is a good environment for human functioning and, second, the model tells us nothing of the features of a community that create resilience. Other researchers have done this: in Table 5 we have listed a number of characteristics attributed to resilient communities across the literature.

**Table 5:** Characteristics attributed to resilient communities

**Characteristic attributed**	**Citation**
Residents of a community have a sense of belonging and orientation to a common purpose	[31,54-56]
Communities have social and organisational networks	[31,41,43,44,57,58]
Communities have access to knowledge and resources, community hope, knowledge promotion skills	[35,44,53]
Communities have strong values on avoiding crime, good parenting, education and work success	[40,53]
Communities with cultural pluralism, inclusivity and social cohesion	[30,41,53,54,56]
Communities with infrastructure and support services	[31,53,56]
Communities have resources and plans that facilitate coping and adaption in adversity	[23,35]
Communities with vibrant participation, shared decision making and collective action	[53,59,60]
Age profile	[53]
Social physical context: physical environment, housing	[53,56]
Population stability, attracting and retaining population	[56]
Facilities and amenities, service provision	[53]

Putting the two identifying models together we can identify the resilient outliers and mark out their characteristics. The difficulty, however, is that in this list of characteristics it is not clear how to distinguish the features that show *that* a community is thriving in difficult times (and is thus resilient) from the features that show *why* it is, what are the mechanisms that trigger resilient responses?

### Fostering resilient communities

Public Health professionals charged with fostering community resilience should proceed with precision and caution. Precision can be aided with the use of the three-question model developed above.

#### Of what?

Public Health professionals generally cover an area and subdivide this into smaller areas or neighbourhoods. A neighbourhood is clearly defined but will often be a community only in a weak sense, lacking bonds of purpose or culture. In developing a neighbourhood’s resilience, therefore, it might be necessary to look within the area for stronger community ties than neighbourhood.

#### To what?

The threats to health and wellbeing can usefully be divided into acute and chronic. Acute threats are usually clear and external to the community; a flu epidemic, for example. Chronic threats are often internal to the community, such as poverty.

#### To what endpoint?

Again, this is reasonably clear with acute threats - a return to normality - but less so with chronic ones, although in general terms we might say that communities and neighbourhoods have the endpoint of providing some of the wherewithal for people to live well or flourish.

However, there are at least two practical political problems with Public Health professionals adopting a Community Resilience approach. The first arises from the internal nature of some chronic threats. For example, one way to improve neighbourhood resilience to poverty would be to remove the poor from the area. Hence one unintended consequence of a focus on neighbourhood resilience to chronic problems is that professionals and local politicians find they can meet their resilience goal by exporting the problem.

The second problem arises from the difficulty distinguishing between the characteristics of wellbeing and resilience. It seems probable that the markers of neighbourhood resilience will be, at best, a subset of the markers of wellbeing and health. Neighbourhood resilience to chronic problems, insofar as it makes sense at all, is likely to be best viewed as an emergent property of the cluster of factors that make a neighbourhood a good place to live. There is already an empirically and theoretically strong literature on this cluster of factors, usually described as social capital or similar.

One response to this might be to claim that the community resilience approach has the advantage of being asset-based. However, the social-capital-style approaches can also be viewed as asset-based. Of particular use here is the five-capital approach [61]. This sets out five types of so-called capital that a community contribute to public health: *human capital* (e.g. skills and education), *social capital* (e.g. social networks), *built capital* (e.g. access to amenities), *natural capital* (e.g. access to green space), and *economic capital* (e.g. income) resources. It is likely that the five types will often co-occur. However, a deprived area, short on economic capital, might be able to compensate for that if it has, for example, good natural or built capital; this would be an asset-based approach.

We noted earlier that neighbourhoods might only be communities in a weak sense. For the notion of community resilience this is a problem as it unclear how you build resilience in these weak communities. A social-capital approach enables policy-makers to change the focus to the broad range of features that make up and strengthen a sense of community rather than on the one-dimensional resilience approach. There is, for example, research on how design principles can foster community within a neighbourhood [62,63].

## Summary

The notion of community resilience has some attraction to those working in Public Health and it is currently popular in policy discussion in the UK. When the notion is examined in detail, however, difficulties emerge. In order for a community to be deemed resilient we need a sense of what a community is for. This can be achieved in theory by reference, for example, to the Capability approach to welfare; however, in practice, it is difficult to separate markers of community resilience from general markers of community wellbeing. This concept of wellbeing has been mapped in both theory and practice, in particular, by reference to ideas such as social capital. We conclude that it is better to focus on these established approaches to community health rather than attempt to build on the vague and modish idea of community resilience.

## Competing interests

The authors declare that they have no competing interests.

## Authors’ contributions

PA, SB and TC performed the literature review that underpins this discussion. PA was the primary author. All authors read and approved the final manuscript.

## Pre-publication history

The pre-publication history for this paper can be accessed here:

http://www.biomedcentral.com/1471-2458/14/62/prepub
